# Exploring the indirect effect of loneliness in the association between problematic use of social networks and cognitive function in Lebanese adolescents

**DOI:** 10.1186/s40359-023-01168-5

**Published:** 2023-05-08

**Authors:** Rami Tarabay, Sarah Gerges, Abir Sarray El Dine, Diana Malaeb, Sahar Obeid, Souheil Hallit, Michel Soufia

**Affiliations:** 1 School of Medicine and Medical Sciences, Holy Spirit University of Kaslik, P.O. Box 446, Jounieh, Lebanon; 2 Department of Biomedical Sciences, School of arts and Sciences, Lebanese International University, Beirut, Lebanon; 3 School of Pharmacy, Lebanese International University, Beirut, Lebanon; 4 College of Pharmacy, Gulf Med University, Ajman, United Arab Emirates; 5 School of Arts and Sciences, Social and Education Sciences Department, Lebanese American University, Jbeil, Lebanon; 6 Applied Science Research Center, Applied Science Private University, Amman, Jordan; 7 Research Department, Psychiatric Hospital of the Cross, P.O. Box 60096, Jal Eddib, Lebanon

**Keywords:** Problematic use of Social Networks, Cognitive function, Adolescents, Loneliness, Lebanon

## Abstract

**Background:**

Problematic use of social networks is a widespread problem that may exert deleterious impacts on cognitive functions. Moreover, studies have added an important link between loneliness and its harmful effect on cognitive functions. Other studies have also revealed that problematic use of social networks among teenagers has a pejorative influence on their social interactions, leading to increased social isolation. Therefore, the goal of our research was to investigate the link between problematic use of social networks and cognitive function in a group of Lebanese adolescents while also taking into consideration the indirect role of loneliness in this relationship.

**Methods:**

This cross-sectional study, which was carried out between January and April 2022, included 379 teenagers (aged between 13 and 17 years), from all Lebanese governorates. The PROCESS SPSS Macro version 3.4, model four was used to compute three pathways. Pathway A determined the regression coefficient for the effect of problematic use of social networks on loneliness; Pathway B examined the association between loneliness and cognitive function, and Pathway C’ estimated the direct effect of problematic use of social networks on cognitive function.

**Results:**

Higher negative social comparison, addictive consequences of problematic use of social networks, and loneliness were significantly associated with worse cognitive function. Loneliness mediated the association between negative social comparison and worse cognitive function, as well as between addictive consequences of problematic use of social networks and worse cognitive function. In addition, higher financial burden was significantly correlated with worse cognitive function, whereas higher physical activity was related to better cognitive function.

**Conclusion:**

In sum, the current study supports that problematic use of social networks is negatively associated with adolescents’ cognitive function, where loneliness seems to play a pivotal role in this equation. The results thus endorse the importance of helping Lebanese adolescents to overcome problematic use of social networks and recover from their loneliness, to achieve a better cognitive/academic performance.

## Introduction

By definition, cognitive functions are the capacities of our brain that allow us in particular to interact with our environment [1]. As such, cognitive function is a broad term that refers to mental processes involved in acquisition of knowledge, manipulation of information, and reasoning [2]. Precisely, the DSM-5 (Diagnostic and Statistical Manual of Mental Disorders-Fifth Edition) characterizes six critical domains of cognitive functions, such as perceptual-motor function, language, executive function, complex attention, social cognition, and learning/memory [3].

Numerous extrinsic factors may however interfere with individuals’ cognitive abilities, causing deteriorations of these functions. For instance, one of these factors is the problematic use of social networks, which has been conceptually defined as a disorder that is not related to a psychoactive substance ingestion while it shares qualities related to a behavioral addiction [4]. Problematic use of social networks encompasses both addiction-related repercussions of social networks use and how these networks may be negatively used in a comparative fashion, without focus on a specific social network [5]. It is considered a widespread problem that may exert deleterious impacts on cognitive functions, such as memory, executive control, visual and auditory attention, and self-control [6]. Indeed, smartphones are a new generation of phones combining communication and entertainment capabilities; they have become so common that the number of mobile cellular subscriptions reached almost 6 billion worldwide in 2020 [7]. Furthermore, 35% of teenagers are major users of electronic media, having online interaction with virtual individuals constantly and throughout the day [8]. With the rapid increase in their use, a new type of health condition known as social media abuse/misuse has recently emerged as a serious public health issue among teenagers [9].

In addition, the relationship between negative social comparisons on social networks and cognitive functions needs to be accounted for [10, 11]. Namely, the temptation to utilize other people as data sources to evaluate one’s own talents and ways of behaving, thinking, or feeling has been termed as “social comparison” [12]. Depending on who the person compares himself/herself to, this comparison might have favorable or bad implications. If the person compares himself/herself to someone regarded as inferior (i.e., downward comparison [13]), his/her self-esteem is promoted; however, when comparing himself/herself to someone considered better (i.e., upward comparison), he/she feels down [14, 15]. In fact, the latter scenario is the classic situation on social networks, which is acknowledged to have aversive effects on mental health [16–19]. Since negative social comparisons on social networks has been shown to be detrimental to mental health and well-being [14, 15], it is conceivable that this condition may pejoratively affect adolescents’ cognitive functioning by provoking anger, envy, and depression [14, 15], and thus, diverting their focus from properly executing cognitive tasks. However, to date, no empirical analysis has examined this hypothesis.

Moreover, according to several studies [6, 20, 21], addictive consequences of problematic use of social networks (PUSN) are linked to impaired cognitive functions [22–24], which can be explained by decreased productivity, communication, and compromise of interactions with others/or new relationships with peers. Research has indeed found that excessive use of social networks in teenagers is likely to be accompanied by significant psychopathological issues, such as cognitive-emotional dysregulation, cognitive impairments, behavioral issues, somatic symptoms, attention deficiencies, depression, and impulsivity [20, 21]. Furthermore, some research has found a clear link between anxiety and ADHD symptoms and pejorative use of social networks [25, 26]; this relationship was explained by the fact that the continuous stimulation provided by social networking sites drastically diminished attention.

On the other hand, solitude and cognitive impairment are two of the factors linked to smartphone addiction [25]. Loneliness is a negative emotion associated with a perceived absence of social connections. A spectrum of serious repercussions on mental and physical health can result from loneliness, which incorporates subjective rather than objective social isolation [27]. Studies have added an important link between loneliness and its harmful effect on cognitive functions [28], explained by the heavy impact of depression, deterioration of acquired skills, and involution of brain development. Other research [29] has revealed that problematic use of social networks among teenagers has a pejorative influence on their social interactions, leading to increased social isolation [30]. In reality, technology has been successfully proved to push people into a condition of false loneliness, according to the literature [28]. As such, there was a link between problematic use of social networks and social isolation, which was explained by the amount of time spent on social networks and the detachment from parents/peers (e.g., lack of communication with parents, constant solitude in their rooms, etc.). Despite having access to technology and virtual connections, the individual is driven into an acute condition of loneliness [31]. In general, the negative repercussions of excessive use of social networks include symptoms such as withdrawal and decreased user productivity, social interactions, physical health, or emotional well-being in everyday life [6]. Thus, there is a possibility that loneliness could be part of the association between problematic use of social networks and cognitive function among adolescents. Moreover, during their quarantine, a big group of adolescents used their cellphones for online classes, also complaining about poor communication/contact with their teachers, which might have influenced their learning/cognitive abilities [32].

Adolescence is certainly an interesting and essential period to study; since cognitive development corresponds to the physiological changes that enable people to think and learn, there is a considerable change during this phase in terms of mentality, decision-making, the construction of connections with parents/siblings, and engagement with the environment [33]. Adolescents are also considered to be more susceptible to developing social media misuse than other age groups, as they seem to be more exposed to technology, including smartphones, online classrooms, and social networks [34]. Consequently, they are more likely to experience problematic use of social networks, social isolation, and cognitive dysfunction.

When it comes to Lebanon, the prevalence of problematic social media use was shown to be 20.2% within the adult population, specifically in young adults aged between 18 and 34 years [35]. Studies have successfully added a better understanding of the high smartphone addiction rate among Lebanese young adults, as well as the impact of smartphone addiction on their self-esteem and temperaments (i.e., mood disorders) [36]. However, there are actually no clear studies in Lebanon that illustrate the association of problematic use of social networks with Lebanese adolescents’ cognitive function. Furthermore, now is the most crucial time to look into this matter because of the COVID-19 pandemic [32]. As a result, there is a pressing need to shed light on the link between problematic use of social networks, cognitive function, and loneliness among adolescents. Consequently, the goal of our research was to investigate the link between problematic use of social networks and cognitive function in a group of Lebanese adolescents, while also taking into consideration the indirect role of loneliness in this relationship. We hypothesized that problematic use of social networks and loneliness would be associated with worse cognitive function among these adolescents; additionally, we expected that loneliness would mediate the relationship between the two components of problematic use of social networks (i.e., negative social comparisons and addictive consequences) and worse cognitive function. In addition, research has successfully shown the influence of sociodemographic variables and personal factors such as the body mass index and physical activity on the relationship between the use of social networks and adolescents’ cognitive functions [37, 38]. To this end, we also sought to control for these confounding variables within this study.

## Methods

### Study design and participants

This cross-sectional study, which was carried out between January and April 2022, included 379 teenagers (aged between 13 and 17 years) who were currently residing in Lebanon and came from every governorate in Lebanon (Beirut, Mount Lebanon, North, South, and Bekaa). The snowball method was used to recruit our sample, Google Forms was used to make a soft copy of the questionnaire, and an online approach was developed to continue with the data collection process. Prior to their participation, participants received online instructions on how to complete the questionnaire as well as the primary aims and objectives of the study. Afterwards, the original participants were requested to find new participants from their networks who were within the same age range and as diverse as feasible in terms of place of residence throughout the Lebanese governorates. There were no remunerative rewards for taking part.

### Minimal sample size calculation

Using the formula proposed by Fritz and MacKinnon [39], to calculate the sample size, n = L/f2 + k + 1, where f = 0.26 for a small to moderate effect size, L = 7.85 for an error of 5%, power = 80%, and taking 10 variables to be included in the model, a minimum sample of 127 was considered required.

### Questionnaire

A questionnaire in Arabic was sent as a Google form link via social media networks. It needed 7–10 min to be filled. It contained sociodemographic information about the participants (age, sex, and governorate of residence). Following the World Health Organization rules of calculation, current self-report weight and height were collected to calculate the Body Mass Index (BMI) [40]. The ratio of the number of people living in the house to the number of rooms in it, known as the household crowding index, was calculated. It reflects the socioeconomic standing of the family [41]. The multiplication of daily exercise frequency, intensity, and duration yielded the physical activity index [42]. On a scale of 1 to 10, with 10 denoting extreme pressure, respondents were asked to rate their feelings of financial strain in relation to their general personal financial condition, answering the following question: “How much pressure do you feel with regard to your personal financial situation in general?”. The second part included the scales used in the current research:

#### Problematic use of Social Networks (PUS) questionnaire

The PUS is a self-report instrument, examining the potential addiction-related repercussions of Social Networks (SNs) use and concentrating on how SNs are used in a comparative way with no restraint to a particular social network, which allows generalization to multiple SNs [5]. The tool is made of 18 Likert-type questions with five response options ranging from 1 = “completely disagree” to 5 = “completely agree”. Statements are categorized into two subscales: 8 questions for addictive consequences of SNs (e.g., “Using social networks, I lose track of time and ignore important tasks I have outstanding”) and 10 questions for negative social comparisons (e.g., “When I see content from influencers or celebrities, I feel inferior”). Greater scores indicate potentiated problematic use of SNs in both domains. In the current study, αCronbach = 0.95 for negative social comparison and αCronbach = 0.90 for addictive consequences.

##### Jong-Gierveld loneliness scale

We assessed subjective loneliness by the modified version of the Jong-Gierveld Loneliness Scale, made of 5 questions (e.g., “I experience a general sense of emptiness”; “I miss having people around”) [43, 44]. A score of 1 is given for a “yes” response and a score of 0 for a “no” answer. Higher ratings reflect increased loneliness. In the current study, αCronbach = 0.76. This scale is validated in Lebanon [45].

#### Cognitive Functioning Self-Assessment Scale (CFSS)

Participants were asked to estimate the frequency of each of the 18 statements on a five-point scale with the “never” and “always” anchors during the course of the previous year. Examples of the 18 statements including “Difficulty in performing two tasks simultaneously” and “Difficulty in performing mental calculation” [46]. Higher scores define lower cognitive function. In this study, αCronbach = 0.95.

## Translation Procedure

On all scales, the forward and backward translation approach was used. A Lebanese translator who had no connection to the study translated the English text from English into Arabic. The Arabic version was then translated back into English by a Lebanese psychologist who is fully functional in English. To identify and then get rid of any inconsistencies, the first and second English versions were compared.

### Statistical analysis

SPSS software version 23 was used to conduct data analysis. Cronbach’s alpha values were computed for each scale and subscale. We had no missing data since all questions were required in the Google form. Cronbach’s alpha values were recorded for reliability analysis of all scales and subscales. The cognitive function score was normally distributed, with its skewness and kurtosis varying between − 1 and + 1 [47].The Student t and ANOVA tests were used to compare two and three or more means respectively, whereas the Pearson correlation test was used to compare two continuous variables. The PROCESS SPSS Macro version 3.4, model four [47] was used to calculate three pathways. Pathway A determined the regression coefficient for the effect of problematic use of social networks on loneliness; Pathway B examined the association between loneliness and cognitive function, and Pathway C’ estimated the direct effect of problematic use of social networks on cognitive function. An indirect effect was deemed significant if the bootstrapped 95% confidence intervals of the indirect pathway AB did not pass by zero. Variables that showed a *p* < 0.25 in the bivariate analysis were entered in the mediation model. Significance was set at a *p* < 0.05.

## Results

### Sociodemographic and other characteristics of the participants

A total of 379 adolescents participated in this study; their mean age was 16.07 ± 1.19 years, with 64.9% females. Table 1 summarizes other characteristics of the participants (Table 1).

**Table 1:** Sociodemographic and other characteristics of the participants (N = 379)

Variable	*N* (%)
Sex	
Male	133 (35.1%)
Female	246 (64.9%)
	**Mean ±** ***SD***
Age (in years)	16.07 ± 1.19
Physical activity index	27.78 ± 20.15
Household crowding index (persons/room)	1.26 ± 0.74
Body Mass Index (kg/m^2^)	22.33 ± 3.79
Financial burden	4.96 ± 2.80
Negative social comparison	20.97 ± 9.73
Addictive consequences of problematic use of social networks	19.53 ± 7.46
Loneliness	2.01 ± 1.73
Cognitive function	25.27 ± 14.22

### Bivariate analysis

Higher negative social comparison (r = 0.55), addictive consequences of problematic use of social networks (r = 0.49), loneliness (r = 0.45), and financial burden (r = 0.27) were significantly associated with worse cognitive function (i.e., higher cognitive function scores), whereas more physical activity (r=-0.24) was significantly associated with better cognitive function (i.e., lower cognitive function scores). No significant difference was found between males and females in terms of cognitive function (24.56 ± 15.70 vs. 25.66 ± 13.38; p = 0.493) (Table 2).

**Table 2:** Bivariate analysis of the continuous variables associated with cognitive function

Variable	CF	NSC	AC	L	Age	PAI	HCI	BMI	FB
Cognitive Function (CF)	1								
Negative Social Comparison (NSC)	0.55^***^	1							
Addictive Consequences (AC)	0.49^***^	0.72^***^	1						
Loneliness (L)	0.45^***^	0.52^***^	0.42^***^	1					
Age	0.05	− 0.05	− 0.01	0.14^**^	1				
Physical activity index (PAI)	− 0.24^***^	− 0.14^**^	− 0.16^**^	− 0.06	0.02	1			
Household crowding index (HCI)	0.02	− 0.09	− 0.09	0.003	0.02	− 0.06	1		
Body Mass Index (BMI)	0.08	0.15^**^	0.11^*^	0.03	0.02	0.03	0.15^**^	1	
Financial burden (FB)	0.27^***^	0.20^***^	0.17^**^	0.25^***^	0.14^**^	− 0.15^**^	0.20^***^	0.11^*^	1

*r* = Pearson correlation coefficient; ^*^p < 0.05; ^**^p < 0.01; ^***^p < 0.001

### Indirect effect analysis

**Fig. 1:**
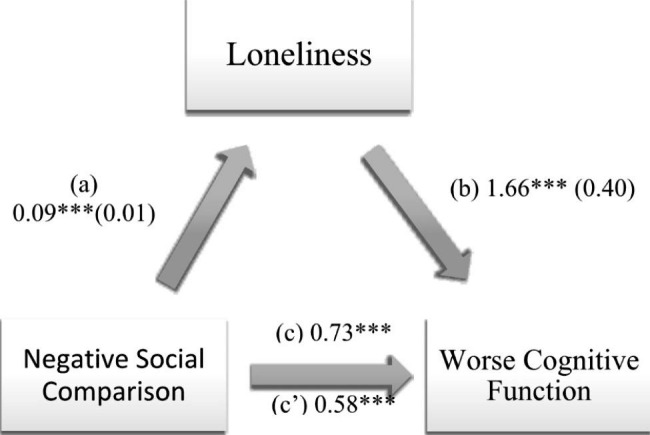
(a) Relation between negative social comparison and loneliness (R^2^ = 29.80%); (b) Relation between loneliness and cognitive function (R^2^ = 37.77%); (c) Total effect of the relation between negative social comparison and cognitive function (R^2^ = 34.90%); (c’) Direct effect of the relation between negative social comparison and cognitive function. Numbers are displayed as regression coefficients (standard error). ***p < 0.001

**Fig. 2:**
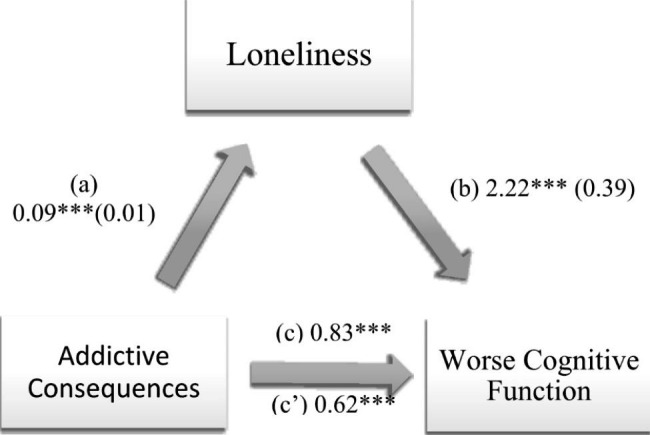
(a) Relation between addictive consequences of problematic use of social networks and loneliness (R^2^ = 29.80%); (b) Relation between loneliness and cognitive function (R^2^ = 37.77%); (c) Total effect of the relation between addictive consequences of problematic use of social networks and cognitive function (R^2^ = 34.90%); (c’) Direct effect of the relation between addictive consequences of problematic use of social networks and cognitive function. Numbers are displayed as regression coefficients (standard error). ***p < 0.001

Loneliness mediated the association between negative social comparison and worse cognitive function, and between addictive consequences of PUSN and worse cognitive function (Table 3; Figs. 1 and 2). Higher PUSN was significantly associated with more loneliness, which in turn was significantly associated with worse cognitive function (i.e., higher cognitive function scores). Finally, higher PUSN was directly significantly associated with worse cognitive function (i.e., higher cognitive function scores).

**Table 3:** Indirect effect analyses results, taking the problematic use of social networks subscales as independent variables, loneliness as the mediator and the cognitive function score as the dependent variable

	Direct Effect			Indirect Effect		
	Beta	*SE*	*P*	Beta	Boot *SE*	Boot *CI*
Negative Social Comparison	0.58	0.07	< 0.001	0.15	0.04	0.07-0.23*
Addictive Consequences	0.62	0.09	< 0.001	0.21	0.05	0.12-0.30*

* indicates significant indirect effect; Beta values refer to unstandardized ones. Direct effect refers to the direct association of PSUN subscale and cognitive function. Indirect effect refers to the association of PSUN subscale and cognitive function through the mediator (= loneliness)

## Discussion

Our study’s objective was to explore both direct and indirect relationships between problematic use of social networks and cognitive function among Lebanese adolescents, taking into account loneliness as a potential actor through the indirect pathway. Our analysis revealed that problematic use of social networks, reflected by addictive consequences and negative social comparison, was strictly associated with worse cognitive function in those adolescents. Moreover, loneliness was identified as a potential mediator of these relationships.

First, our results have indeed demonstrated the positive link between the addictive consequences of problematic use of social networks and inferior self-reported cognitive functioning in Lebanese adolescents. Certainly, by the second decade of life, the brain’s structure is considerably modulated, with a reduction of gray matter, increase in while matter, and thus long-term effects on human cognitive function [48–50]. Such structural modulations have actually shown remarkable dependency to external/environmental factors, such as traumatic childhood experiences or the child’s raising medium [51, 52]. Moreover, social media use disorders (specifically addictive consequences) have also been found to be critical in determining cognitive function among adolescents. To exemplify, Wang et al. revealed that adolescents who endorsed internet addiction had lower levels of gray matter across the bilateral cerebellum and a variety of brain regions, as well as alterations in cognitive control [50]. Additionally, through a functional neuroimaging study, Li et al. have evidenced that adolescents with addictive behaviors to internet suffered from impairments in frontal-basal ganglia connectivity, with those dysfunctions severely interfering with their cognitive ability to inhibit undesirable behaviors [53]. Consistently, Horowitz-Kraus and Hutton have discovered the presence of lower levels of functional brain connectivity in basically healthy children who had an increased time spent on screen-based media; specifically, increased exposure to screens was correlated with a diminished connectivity between the left visual word form area and brain areas responsible for language and cognitive regulation [54].

In addition, our study highlighted that negative social comparisons in relation to the content seen on social networks was significantly associated with worse cognitive function in Lebanese adolescents. In line with this perspective, it has previously been documented that social comparisons and depressive moods may exert a significant impact on adolescents’ social decision-making, which represent an important negative repercussion on their cognitive functioning [55]. As stated in the introduction, when comparing themselves to other people or celebrities on social networks, adolescents tend to feel down and unworthy [14, 15]; this negative comparison on social networks then yields deleterious effects on their mental health and abilities to concentrate on other important tasks.

Further, numerous studies have shown that problematic use of social networks among adolescents engenders a wide array of psychopathological symptoms, prominently loneliness [21, 56, 57]. On the other hand, loneliness and depressive symptoms appeared to be incriminated in lowering cognitive function among older adults [58–60], with a scarcity of data covering this relationship among younger age groups. However, some research has in fact highlighted the deleterious effects of social deprivation on adolescents’ development and mental health [61, 62], as adolescence is a life period outlined by an elevated need for interaction with peers and an enhanced susceptibility to social stimuli and to the nocive repercussions of social exclusion [63]. For instance, both peer social acceptance and peer influence are crucial aspects of development in adolescence [63], where social deprivation exerts unique adverse effects on adolescents’ brain and behaviors in comparison with other age groups [61, 64–66]. The abovementioned facts thus sustain the potential indirect effect of loneliness, demonstrated by the present study, when studying the relationship between problematic use of social networks and cognitive function in adolescents, and call for more studies in this research area among this vulnerable age group.

Lastly, our analysis also showed that physical activity was related to higher levels of cognitive functioning, whereas adolescents having a higher financial burden had worse cognitive function. Those findings are consistent with previous literature showing that moderate physical activity bolsters cognitive function as well as brain’s structure and function among adolescents [67, 68], primarily by enhancing general health and subjective well-being [67]. On the other hand, studies have shown that the socio-economic status is linked to adolescents’ cognitive competencies, namely language development and executive functions, with socially disadvantaged adolescents having deteriorated cognitive processes [69]. Indeed, disparities within adolescents’ education, which are highly dependent on their socio-economic level in Lebanon (a developing country), could support and explain our finding.

### Clinical implications

The results of this study may benefit school counselors, teachers, and parents, who should remain vigilant towards adolescents who lack true friendships or seem lonely. They should also be mindful of the possibility of problematic use of social networks underlying adolescents’ social isolation and thus implement effective awareness programs and supportive measures for those adolescents (e.g., social media guidance, social media time management, engagement in outdoor activities, promotion of physical activity, etc.), in order to help them limit their social media exposure, open up, and establish healthy and solid relationships with their peers. Detecting and fixing such problematic behaviors at a precocious level is very important to prevent deterioration of cognitive function among adolescents and protect their mental health and academic performance. As such, clinical psychologists and school psychologists are prompted to look into these matters in a dependent way and consider the possibility of their interrelations, in order to better identify the origins of cognitive function decline among adolescents and establish proficient and integral treatment and management plans. Specifically, this study might advance several recommendations, such as implementing leisure services other than smartphones for adolescents/students, facilitating adolescents’ access to sports and other free activities in schools, designing awareness lectures and programs about the deleterious impacts of problematic use of social networks among students, and enacting policies to restrain and supervise their use of smartphones and social networks. Future research might also build on our results to identify other factors that might play a role in the relationship between problematic use of social networks and cognitive function in adolescents.

#### Limitations

Caution is essential when interpreting the findings and conclusions generated by this study, taking into consideration several limitations. First, its cross-sectional design is unable to infer causality and temporality of the studied correlations. A selection bias might also have occurred, owing to the fact that the data was derived from a non-probability sample using the snowball sampling method via online networks. However, this sampling technique was the only available option during the COVID-19 pandemic and its related security measures. In this context, an information bias is possible since the study relied on self-report measures rather than a physician’s assessment. A residual confounding bias is possible as well, as some additional predictors of the studied relationships might have been overlooked. Finally, it is important to mention that we could not vigorously assess the construct of loneliness, which could be emotional/intimate, social, or collective. Some adolescents might have more online interactions with their friends on social networks, but still feel lonely. The subjectivity regarding the definition and experience of loneliness should be taken into account and further investigated in the relationship between problematic use of social networks and cognitive function in upcoming research.

## Conclusion

In sum, the current study supports that problematic use of social networks is negatively associated with adolescents’ cognitive function, where loneliness seems to play a pivotal role in this equation. The results thus endorse the importance of helping Lebanese adolescents to overcome problematic use of social networks and recover from their loneliness, to achieve a better cognitive/academic performance. Future investigations into additional comprehensive factors contributing to the relationship between problematic use of social networks and cognitive function in adolescents may help to further understand its complexity for implementing better interventions.

## Data Availability

All data generated or analysed during this study are not publicly available to maintain the privacy of the individuals’ identities. The dataset supporting the conclusions is available upon request to the corresponding author.
